# Slow-growing and Linearly Spreading Cutaneous Lesion: Often Misdiagnosed Mycobacterium Marinum Infection

**DOI:** 10.7759/cureus.4154

**Published:** 2019-02-28

**Authors:** Lakshmisree A Vemulakonda, Jamie A Tschen

**Affiliations:** 1 Pathology, University of Washington, Bothell, USA; 2 Dermatopathology, St. Joseph Hospital, Houston, USA

**Keywords:** mycobacterium marinum, fish tank granuloma, sporotrichoid spread

## Abstract

*Mycobacterium marinum* is a slow-growing atypical mycobacterium. It is a photochromogen; when exposed to light, it produces yellow pigment. In humans, it manifests as a localized granuloma or sporotrichotic lymphangitis. Patients at risk include anglers (commercial, recreational), oyster workers, swimmers, aquarium workers, and individuals with aquariums in their homes. Herein, we report a case of a *Mycobacterium marinum* infection which was misdiagnosed because there was no histopathological evidence of acid-fast bacilli and the slow growth rate in cultures.

## Introduction

*Mycobacterium marinum* is an atypical mycobacterium that causes dermatological and osteoarticular lesions. The main source of infection is exposure to the aquatic environment or marine life. Despite an increase in the number of cases in recent years, this infection often goes unrecognized or misdiagnosed due to the non-specific clinical and histopathological features and the slow rate of growth in cultures leading to delayed management [1]. In this case report, we would like to familiarize the reader with this infection and its management to minimize the risk of misdiagnosis and delayed treatment.

## Case presentation

**Figure 1:**
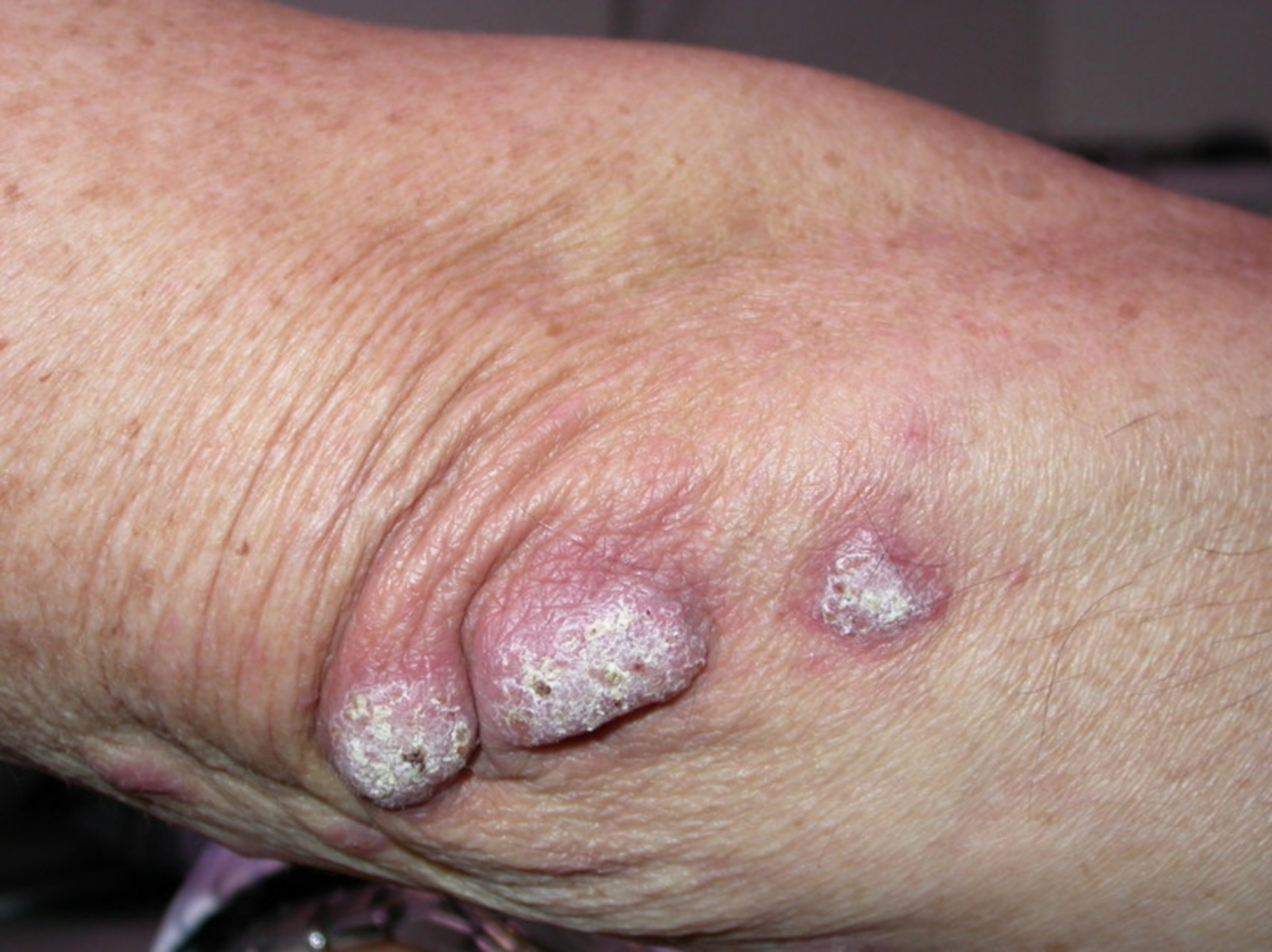
Three verrucous plaques on the right elbow of the patient

**Figure 2:**
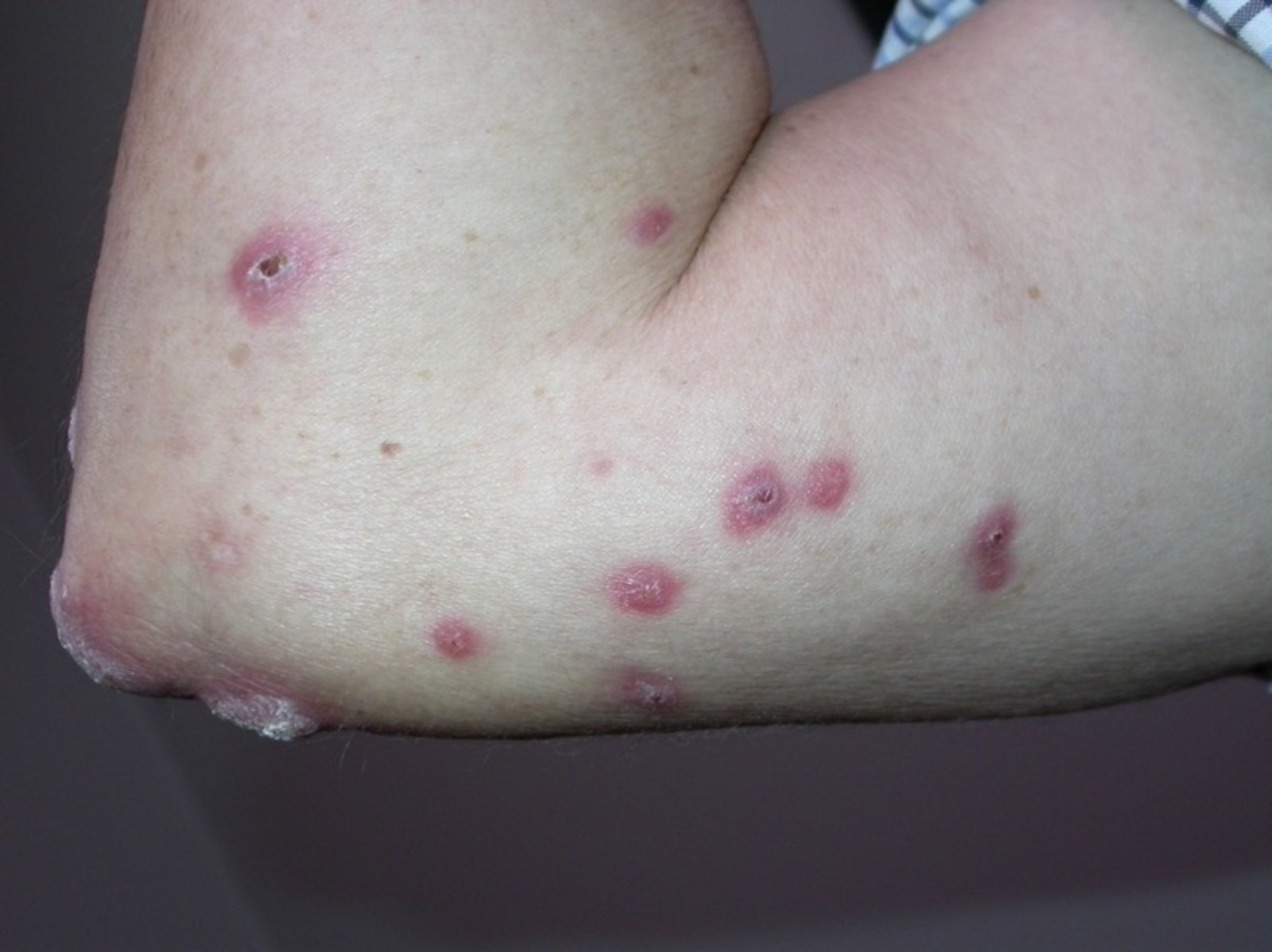
Ulcerated papulonodules on the medial aspect of the right elbow of the patient

A 69-year-old male came with a complaint of cutaneous lesions on the right arm associated with redness and itching for 11 months. Other skin lesions and systemic symptoms were notably absent. A physical examination revealed three verrucous plaques on the right elbow and ulcerated papulonodules on the medial aspect of the right elbow (Figures 1-2).

**Figure 3:**
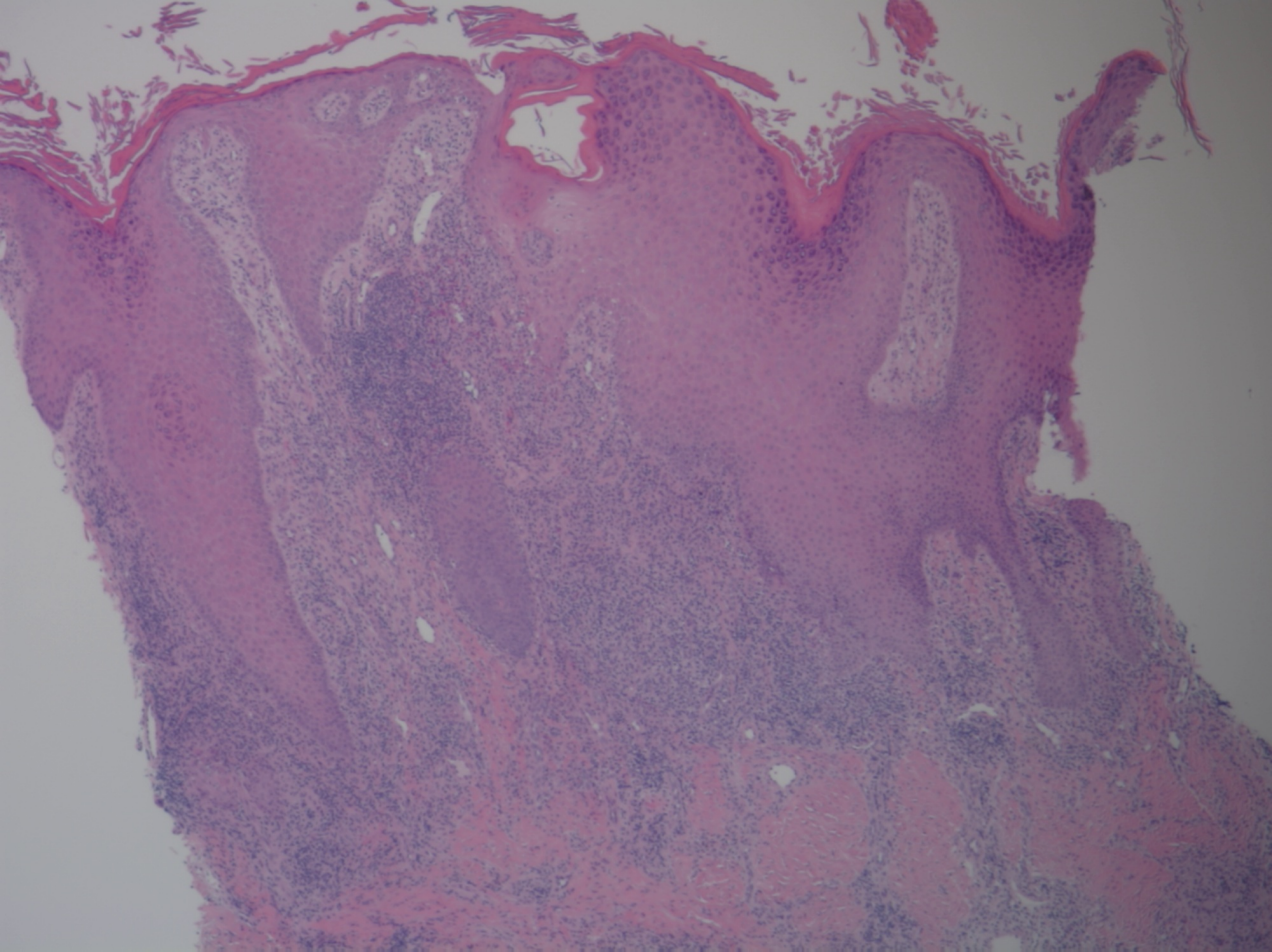
Histopathological examination in low power showing acanthosis, hyperkeratosis, and chronic granulomatous inflammation

**Figure 4:**
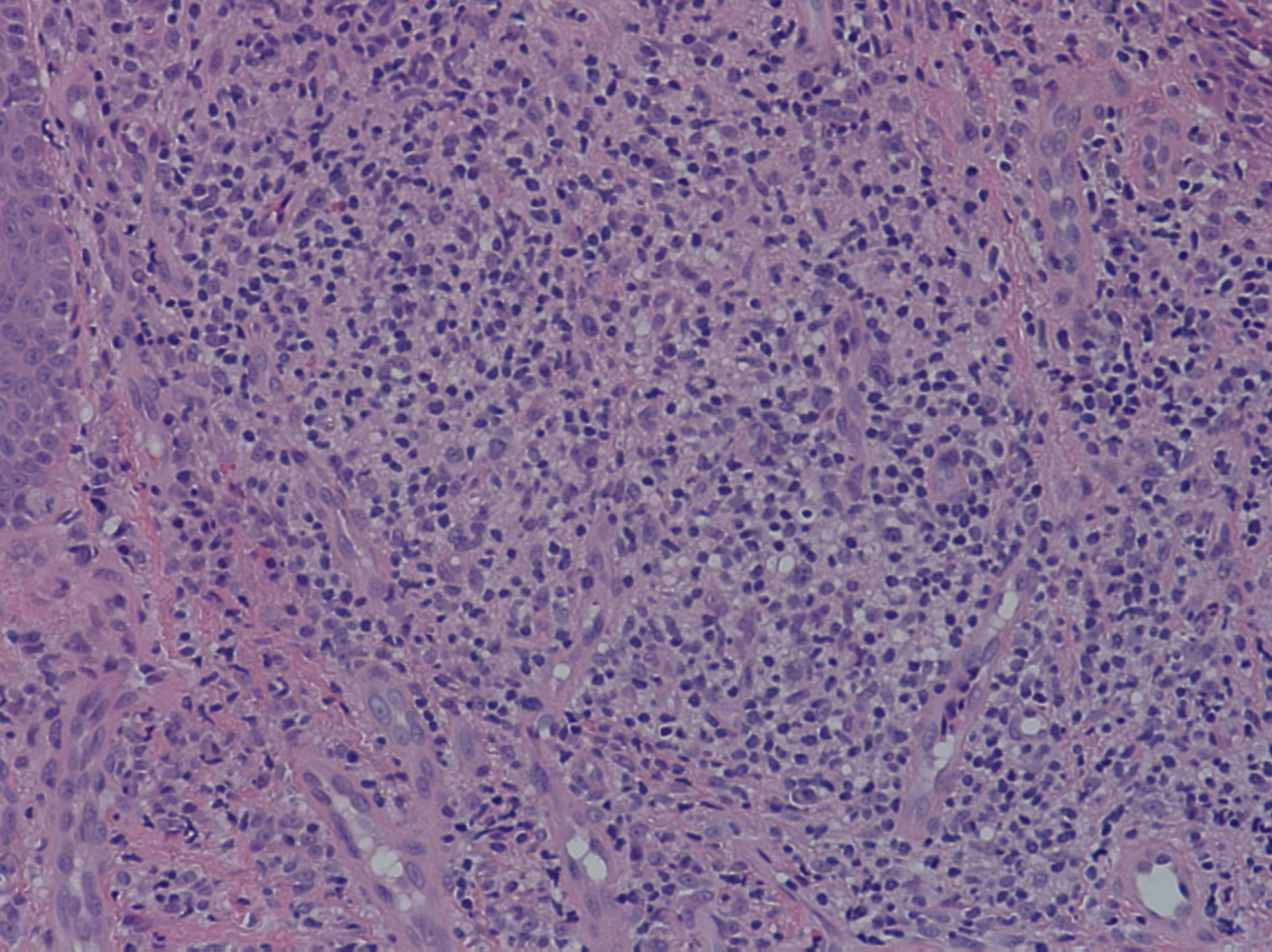
Histopathological examination in high power showing diffuse mixed inflammatory infiltrate in the dermis

A 4 mm punch biopsy from the medial aspect of the right elbow demonstrated chronic granulomatous inflammation with diffuse dermal mixed infiltrate of neutrophils, histiocytes, and plasma cells and occasional microabscesses (Figures 3-4). Gram stain, acid-fast bacilli (AFB), and periodic acid-Schiff (PAS) stains with appropriate controls for organisms were negative. Cultures obtained were negative for fungus and AFB at six weeks.

A diagnosis of sporotrichosis was made based on the sporortrichoid spread of lesions in a linear pattern up the lymphatics, histopathological findings, and the absence of AFB on special stains and culture. The patient was started on itraconazole.

After a month, the patient came back for a follow-up with a flare-up of the lesions on the right elbow. On probing, the patient admitted to cleaning an aquarium at home before the start of lesions. A biopsy of the lesions was sent for AFB stain, PAS stain, and culture for fungus and AFB. The fungal stain showed no yeast and hyphae. No AFB were found on direct smear. No fungus was isolated at six weeks of culture. At seven weeks of culture, AFB was isolated and identified as *Mycobacterium marinum* (*M. marinum*). The patient was started on rifampin, 300 mg twice a day, and ethambutol, 400 mg five times a day. The patient reported considerable improvement with the above treatment.

## Discussion

*M. marinum* is a non-motile, non-spore forming, slow-growing photochromatic AFB. It produces a yellow pigment when exposed to light. *M. marinum* was reported as the most common cutaneous, nontuberculous mycobacterial infection with an incidence of 0.04 to 0.27 per 100,000 inhabitants [2]. The first human case of *M. marinum* isolated from skin lesions of swimmers who bathed in a contaminated pool in Sweden was reported by Norden and Linell in 1951 [3].

*M. marinum* infection, also known as aquarium granuloma, swimming pool granuloma, or fish tank granuloma, can be contracted through minor abrasions incurred while bathing in swimming pools, ocean, or lake water or while cleaning home aquariums. A thorough history of the disease with a review of occupational and background exposure to potential pathogens during recent travels and leisure activities will help in the diagnosis of the infection.

*M. marinum* can clinically present as painful or painless, solitary or multiple erythematous papules, nodules, plaques, or verrucous lesions with or without crust or ulceration. In some instances, a sporotrichoid spread without accompanying lymphadenopathy has been reported [4]. The lesions can be categorized into three types to guide the treatment: 

▪ Type 1 lesions: these are 1 - 2 cm superficial erythematous popular or verrucous self-limited lesions. Treatment with antibiotics may be required if remission is not observed.

▪ Type 2 lesions: these are subcutaneous granulomas that require treatment with antibiotics.

▪ Type 3 lesions: these are lesions that involve deeper structures, like tendons, bones, and joints, and require surgical debridement, along with antibiotic therapy [5].

Histopathological findings can range from a non-specific inflammatory infiltrate composed of neutrophils, monocytes, and macrophages to tuberculoid granulomas depending on the duration of the lesion. Hyperplastic or ulcerated epidermis with microabscesses can be found. Acid-fast stains may reveal organisms, but the sensitivity of acid-fast stains on biopsy material is low.

A culture of the involved tissue is crucial for establishing the diagnosis of *M. marinum* infection. The culture should be obtained even in the absence of microscopic evidence of bacilli, as the lesions have a very low concentration of microorganisms. The laboratory should be notified that *M. marinum* infection is suspected. Cultures are incubated in Lowenstein-Jensen agar at 28° to 32° C (in addition to 37° C) and observed for six to 12 weeks [6]. The positivity rate of culture ranges from 70% to 80%; hence, as illustrated in the presented case, organisms may not be recovered from the first culture performed and additional biopsy material for culture may be needed [7].

Recent advances in molecular methods allow definitive and rapid identification of *M. marinum*. Polymerase chain reaction (PCR) amplification techniques using mycobacterium genus-specific primers can be used to diagnose an *M. marinum* infection directly in the biopsy sample. In-solution hybridization or solid format reverse hybridization assays enable identification in the early stage of bacterial growth [8].

Management of the infection depends on the severity of the lesions. Early lesions are usually self-limited. They can resolve spontaneously or with antibiotic monotherapy, such as clarithromycin, azithromycin, moxifloxacin, tetracyclines, or trimethoprim/sulfamethoxazole. Severe infections with a sporotrichoid distribution or extensive infections with deeper structure involvement require multidrug therapy with rifampicin and ethambutol or clarithromycin in association with rifampicin or ethambutol. The duration of treatment may vary between six weeks to six months with treatment often continuing one to two months after the clinical remission. Surgical debridement is required in cases with subcutaneous tissue involvement [9].

## Conclusions

In summary, the present report describes a case of an *M. marinum* infection in a 69-year-old immunocompetent male with cutaneous lesions on the right elbow for 12 months. Histopathology detected chronic granulomatous inflammation with no evidence of fungi or AFB. The culture, in an appropriate medium, confirmed the diagnosis and treatment with rifampicin and ethambutol was effective.

The diagnosis and management of an *M. marinum* infection are difficult without a proper history and a high index of suspicion for the disease. Patients presenting with indolent nodular skin infections affecting the upper extremities should be questioned about aquatic exposure. Tissue biopsy for histopathologic examination and culture in the appropriate medium, even in the absence of AFB in special stains, is essential to establish an early diagnosis and promptly initiate the appropriate therapy.
